# Do Immature Defense Mechanisms Mediate the Relationship Between Shame, Guilt, and Psychopathological Symptoms?

**DOI:** 10.3389/fpsyg.2022.832237

**Published:** 2022-05-03

**Authors:** Cesare Cavalera, Paolo Andreani, Oliver Baumgartner, Osmano Oasi

**Affiliations:** Deparment of Psychology, Università Cattolica del Sacro Cuore, Milan, Italy

**Keywords:** defense mechanism, shame, guilt, symptoms severity, self-conscious emotions

## Abstract

When shame and guilt emotional experiences related to stressful events remain unresolved, they can be related to psychological impairment and recursive thoughts. The present study aims to explore the association between state shame and state guilt related to past stressful experiences and psychopathological symptoms and evaluating a mediation role by immature defenses. A total of 90 participants (48.9% female; mean age 23.66) were considered in the present study to (a) investigate correlations between state guilt and shame scores related to personal stressful events and psychopathological symptoms related to global severity index; (b) assess whether state guilt and shame scores related to personal stressful events are positively correlated with immature defenses; (c) test whether immature defenses mediates the relationship between, respectively, state shame and state guilt with psychopathological symptoms. Significant correlations between state shame, state guilt, psychopathological symptoms, and immature defenses were found. Higher activations on immature defenses partially mediated the relationship between psychopathological symptoms and state shame and state guilt, respectively. Past experiences related to shame and guilt should be targeted by specific treatments that could help stop recursive maladaptive thoughts and empower more adaptive defensive strategies.

## Introduction

### Traumatic and Psychopathological Implications of Shame and Guilt

Trauma and memories of trauma can be related to unresolved negative emotions with serious consequences for quality of life and psychological balance (Vaillant, 1994; Dorahy et al., 2017). Guilt and shame are distinctive negative self-conscious emotions that, due to their nature, can be related to traumatic experiences (American Psychiatric Association, 2013; Cavalera et al., 2018). Despite the fact they have many aspects in common (i.e., both originate from relevant transgression and failure of oneself), they differ in their trajectories. While guilt implies self-criticism of a specific personal action and it is associated with the damage it may have caused to others, shame is the consequence of a negative self-evaluation (Tracy and Robins, 2006). While feeling of shame are linked to a stable internal and negative perception of the entire self (e.g., “I’m a bad person”), guilt appears when people attribute an adverse outcome to internal, unstable, and controllable attributions (e.g., “I’ve made a bad mistake”). Therefore, shame is associated with feelings of inferiority and worthlessness for a defective self with the consequent desire to hide or escape from a bad situation, while guilt is associated with feelings of tension and remorse related to a wrong behavior (Tracy and Robins, 2006). As shame is intrinsic to social subordination, recursive shameful experiences may be related to thoughts that reflect a defective self. Maladaptive guilt may be also related to impairing thoughts of hyper-responsibility and self-criticism that occurs in interpersonal situation (Cavalera, 2020). Therefore, when shame and guilt related to stressful event remain unresolved, they can readily be elicited in everyday situations causing psychological impairment and recursive thoughts (Irwin, 1998; Dorahy et al., 2017).

Different studies explored the association between stressful experiences, shame, and guilt considering them as negative state emotional experiences on one side or as lasting affective personal predisposition on the other side. Within the first perspective shame and guilt are analyzed as state emotions that are elicited by specific and contextual social situations and that are assessed by exploring how the participant is feeling in the here and now (Cavalera et al., 2017). As shame and guilt not only consist in transient emotional experiences, a second and integrative view analyzed them in terms of lasting affective predisposition using for example scenario-based measures (Tangney and Dearing, 2003). Within this last perspective, a lot of studies evidenced a relationship between shame and guilt proneness and psychopathological symptoms that can be associated with unresolved traumatic experiences (Tangney and Dearing, 2003; Vikan et al., 2010; Vizin et al., 2016). While in most of the case the relationship between shame and psychological symptoms was confirmed (Rüsch et al., 2007; Schoenleber and Berenbaum, 2012), the relationship between guilt and symptoms severity is characterized by more controversial results (Harder et al., 1992; Tangney et al., 1992; Averill et al., 2002; Fergus et al., 2010; Vizin et al., 2016). Moreover, when the impact of shame and guilt on Post-Traumatic Stress Disorder (PTSD) symptoms are examined together, shame typically emerges as the strongest predictor (e.g., Street and Arias, 2001). Leskela et al. (2002), exploring PTSD in 107 former prisoners of war, found that shame-proneness but not guilt proneness was related to severity of psychological symptoms. Once the effects of shame were statistically removed, guilt showed a negative relationship with psychological symptoms. This suggests that shame rather than guilt is central to the pathognomonic outcome of relational stressful experiences. However, all these previous studies explored the role of shame and guilt proneness without analyzing the association between symptoms and state shame and guilt specifically related to personal stressful events.

Besides shame and guilt proneness, state shame and state guilt considered as negative emotional experiences can both be related to very stressful psychopathological symptoms. Recent studies explored shame and guilt relations with impairing working memory process (Cavalera et al., 2018) and with emotional dysregulation defined both in terms of overwhelming sensations and in terms of excessive deactivation (Rugens and Terhune, 2013; Dorahy et al., 2017). Unresolved state guilt or shame related to traumatic experiences may therefore cause panic, terror, or a sense of urgency (Breggin, 2015). On the other hand, excessive deactivation of emotions may cause depersonalization and derealization, or dissociative emotional numbing in such contexts that intensified emotions would be expected to occur (Pur, 2014). Rugens and Terhune (2013) evidenced the role of guilt in augmenting the relationship between dissociative tendencies and state dissociation. Similarly, State shame was also found to be a significant predictor of dissociative symptoms in complex PTSD (Dorahy et al., 2017).

### Shame, Guilt, and Immature Defenses

Defense mechanisms play a central role in mitigating the distressing effects of emotions and mental representations that are generally associated with conflict (Boldrini et al., 2020). Defense mechanisms are categorized and assessed on a continuum from adaptive to pathological. More adaptive defenses (e.g., humor, altruism, sublimation, and suppression) usually maximize the awareness of affective mental states, allowing an individual to adaptively modulate the expression and gratification of personal needs and desires (Boldrini et al., 2020). Conversely, less adaptive or immature defenses (e.g., denial, acting out, projective identification, and splitting) may involve distortions in the internal representations of self, others, or external reality, and keep potentially threatening ideas, feelings, memories, wishes or fears out of one’s awareness (Maffei et al., 1995; Boldrini et al., 2020).

Emotional dysregulation related to unresolved shame and guilt experiences can lead to the use of involuntary immature defenses (i.e., acting out, splitting, displacement, and dissociation) that are commonly associated with less adaptive functioning (Prunas et al., 2019). The tendency to experience immature defense is characterized by disruptions between the normally integrated systems of attention, awareness, memory, and identity (Dalenberg et al., 2012). There is evidence that negative emotional states, such as guilt and shame, may mediate this relationship between immature defenses, such as peritraumatic dissociation and trait dissociation (Barnow et al., 2011; Rugens and Terhune, 2013). More specifically, the experience of guilt is a reliable predictor of immature defenses, which may function as a coping mechanism following trauma (Spiegel, 1986; Irwin, 1998). For example, considering dissociative responses Rugens and Terhune (2013) found that those participants who had a trait propensity toward dissociation reported a higher dissociation score immediately after exposure to guilt cues than after exposure to general negative cues and neutral cues. The present mechanism may lead to affect regulation, reducing the emotional impact of guilty feelings by creating psychological distance *via* strategies, such as depersonalization, derealization, intense absorption in selected stimuli, confusion about oneself, losing self-reference, and amnesia (e.g., Platt and Freyd, 2015). In the same way, many years of studies on the association between shame, dissociative tendencies, and abuse evidenced that immature defenses and shame are consistently related (Talbot et al., 2004; Dorahy et al., 2017; Khosravi, 2020); more specifically, Dorahy et al. (2017) found out that shame-evoking stimuli have the capacity to produce heightened initial intrusions, and dissociation as a consequence of this shame appears to be a driver in the development of more distressing intrusions. These findings add support to the importance of exploring the role of state shame and guilt and immature defenses related to psychological symptoms in a more broader perspective and not considering only dissociative defenses (Andrews et al., 1989; Leskela et al., 2002; Aakvaag et al., 2014).

### Immature Defenses and Psychopathological Risk

Growing evidence confirmed the association between the use of primitive and dysfunctional defense mechanisms and the psychopathological risk (Zanarini et al., 2009; Calati et al., 2010; Marchesi et al., 2011). Studies in both clinical (Spinhoven and Kooiman, 1997; Calati et al., 2010), and non-clinical samples highlighted the link between immature defenses and psychological symptoms even for sub-clinical form of distress (Muris et al., 2003; Cramer, 2006; Sarno et al., 2010). Spinhoven and Kooiman (1997) evidenced that compared to controls, anxiety and depressive disorder patients scored higher for the use of immature defense style. More recently, Prunas et al. (2019) confirmed the association between the use of primitive defenses and the psychopathological risk with a moderating role of gender showing that women obtained higher scores than men. In a more recent study, immature defenses showed correlations with insecure attachment in a non-clinical sample of adults (Prunas et al., 2019). Hyphantis (2010) evidenced that the use of immature defenses increased the variance explained of 19.2% related to global severity index scores. While adaptive defenses showed negative correlations with GSI, maladaptive defenses were the major independent correlate of global severity index (Hyphantis, 2010).

Although defense mechanisms are often directed against threats to self-esteem to protect the self from the negative effect of disappointment (Cramer, 2006), no study so far explored the role of immature defenses in the relation between negative self-conscious emotions and symptoms severity.

### The Current Study

The current study explored the link between unresolved negative self-conscious emotions, defenses, and psychopathology. As growing empirical evidence confirmed the close association between defense style, unresolved negative emotions and mental health (Andrews et al., 1989; Spinhoven and Kooiman, 1997; Bond and Perry, 2004; Prunas et al., 2006), the aim of the present study is to explore the relationship between state guilt and state shame activations related to personal stressful experiences and to evaluate their association with psychopathological symptoms. In particular, we will focus on the investigation of the relationship between shame and guilt activations, symptoms severity, and immature defenses, evaluating a mediation model. In line with these aims, specific hypotheses were: (1) State guilt and shame scores related to personal stressful events are positively correlated with psychopathological symptoms related to global severity index; (2) State guilt and shame scores related to personal stressful events are positively correlated with immature defenses; (3) Immature defenses mediate the relationship between, respectively, state shame and state guilt with psychopathological symptoms.

## Materials and Methods

### Participants

Participants at least 18 years of age were recruited through the main social networks related to Catholic University of Milan. All the participants provided informed consent after receiving a complete description of the study and had the opportunity to ask questions before completing the self-report questionnaires *via* the Qualtrics online platform. No incentives were provided to the participants. The present study was approved by the ethics board of the Catholic University of Milan. In the course of the recruitment, a total of 140 participants were reached out to complete the study. When participants provided incomplete responses regarding the variables of interest data were excluded by the analysis.

Because of missing data when completing the assessment, 50 subjects were excluded from the analysis. A possible explanation of such a high number of incomplete answers is that some of the participants probably thought they could take a break and resume the questionnaire later only to discover when they returned that they had to start over again. The final group consisted of 90 participants (48.9% female). Mean age was 23.66 (*SD* = 2.14, range 19–28).

### Measures

**Symptom Checklist-90** (SCL-90, Derogatis et al., 1973) is a self-administered questionnaire consisting of 90 items on the sensations experienced in the week prior to administration. The questionnaire has a Likert scale from 0 (not at all) to 4 (very much). There are 10 dimensions relating to clinical psychopathological symptoms, and it is possible to obtain the Global Severity Index (GSI). For the purpose of the present study, only GSI was considered. The scale showed a good index of internal consistency and reliability with values ranging from 0.73 (Rosen et al., 2000) to 0.85 (Donini et al., 2020).

**Response Evaluation Measure** (REM-71, Prunas et al., 2006) is a self-report scale composed of 71 items that investigates 21 defenses on a 9-step Likert scale 1 (strongly disagree) 9 (strongly agree). There are two factors related to adaptive (composed by 7 scales) and immature defense mechanisms (composed by 14 scales). For the purpose of the present study, only immature defense factor was considered. The Italian version showed a good internal consistency, with a value of 0.82 for the immature defenses and 0.72 for the mature defenses (Prunas et al., 2019).

**State shame and guilt scale-8** (SSGS-8, Cavalera et al., 2017) is a self-reported questionnaire consisting of 8 questions based on a Likert-type scale to 5 from 1 (disagree) to 5 (very agree) that explore state guilt and shame. Examples of shame items are “I feel small,” “I feel worthless, powerless,” while examples of guilt items are “I feel tension about something I have done,” “I feel like apologizing, confessing.” SSGS-8 showed a good reliability for both the guilt (Cronbach *α* = 0.84) and shame (Cronbach *α* = 0.81) factors (Cavalera et al., 2018).

### Procedure

After reading and accepting the informed consent, participants completed an initial session of socio-demographic questions. Next, an autobiographical emotional memory task in which subjects were asked to report the most unpleasant experiences they experienced was administered. They were given the following instructions: “Take a few minutes and think back to the three most unpleasant events you have ever experienced. For each of these three events, identify the worst scene and report it below. Write freely without worrying about mistakes. When finished, you can do whatever you want with this sheet. What you write will not be read by anyone.” Later, participants were asked to complete the SGSS-8 in relation to each of the reported stressful experiences. After that SCL-90 and REM-71 were administered. At the end of the writing emotional task, participants were reported that a psychologist of the research team was available for eventual psychological support. In the event that the re-enactment elicits a high level of stress, the contact details of an adequately trained researcher were provided at the end of the experiment.

### Statistical Analyses

Statistical analyses were conducted using IBM SPSS, version 22.0. The within-group Mahalanobis distance showed that considering the variables of interest there were no multivariate outliers (Mahalanobis distance *p* < 0.001; Tabachnick et al., 2007). Means and standard deviations were calculated for each continuous variables measured in the study. Comparisons were made using Pearson correlation. As some of the data were not normally distributed (GSI and state shame scores), for correlations in which at least one of the variables was not normally distributed Spearman was used instead of Pearson analyses according to the distribution of the variables (Mishra et al., 2019).

Finally, mediation models were tested using the SPSS macro PROCESS (Hayes, 2013). The indirect effects of, respectively, state shame, and state guilt on psychopathological symptoms through immature defenses were analyzed through two separated mediation models. Direct and indirect effects were estimated using Preacher and Hayes techniques with 5,000 bootstrap samples (Preacher and Hayes, 2004). Mediation effects were further evaluated using bias-corrected bootstrap 95% confidence intervals (CI). The effects were considered statistically significant if the confidence intervals did not contain zero.

## Results

Descriptive statistics, including demographic information, means, standard deviations, and ranges for the considered outcomes at T1 are reported in Table 1.

**Table 1:** Participants characteristics.

Variables	Range	Mean	*SD*
Age	19–28	23.66	2.14
State guilt	12–46	26.52	9.22
State shame	13–58	33.16	8.98
Psychopathological symptoms	0.01–2.27	0.89	0.46
Immature defenses	8.64–17.71	12.40	2.21

*N = 90.*

In terms of years of education, the sample showed a rather high level as 33.3% reported having attended college while 45.6% reported having bachelor’s degree and 14.4% master’s degree. More information related to other demographic measured and the descriptive statistics divided for male and females were provided in the supplementary material (Supplementary Tables S1, S2).

Pearson and Spearman correlations between variables are reported in Table 2. All the correlations between variables were significant and were in the expected direction. The strongest correlations were between psychopathological symptoms and the other variables of interests confirming the initial hypotheses. More specifically state guilt and shame scores related to personal stressful events were positively correlated with psychopathological symptoms related to psychopathological symptoms and with immature defenses.

**Table 2:** Correlation between study variables.

Variables	1	2	3	4
State guilt	1	0.33^a^^,^ ^**^	0.47^a^^,^ ^***^	0.24^a^^,^ ^*^
State shame		1	0.36^a^^,^ ^**^	0.21^b^^,^ ^*^
Psychopathological symptoms			1	0.59^a^^,^ ^***^
Immature defenses				1

*N = 90.*

* *p* < 0.05;

** *p* < 0.01;

*** *p* < 0.001.

a Spearman rho.

b Pearson *r.*

The parallel mediation model was used to evaluate separately the indirect effect of state shame and state guilt on psychopathological symptoms through the effect of immature defenses. Bootstrapped confidence intervals showed that both the associations between state shame and GSI and between state guilt and GSI were partially mediated by immature defenses.

**Figure 1:**
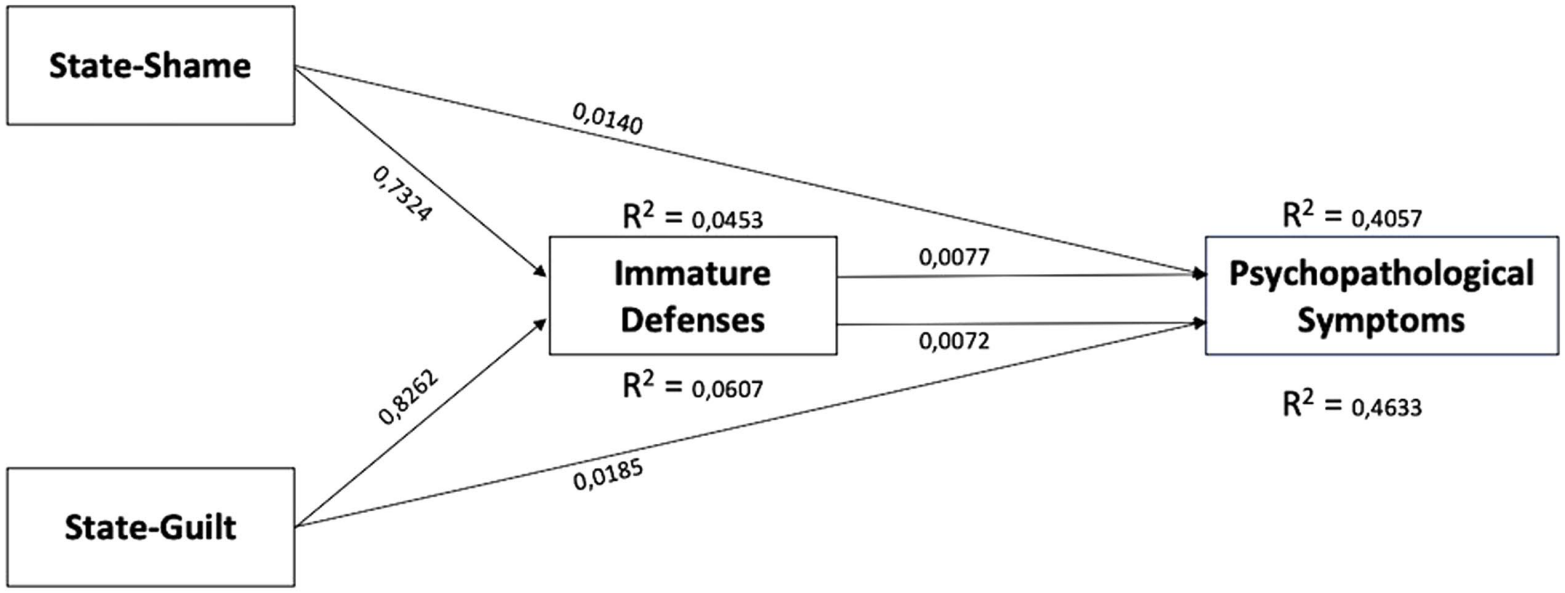
Mediation model of the mediating role of immature defenses in the relationship between state-shame, state-guilt, and psychopathological symptoms.

The overall model related to state guilt accounted for 46.33% of the variance in psychopathological symptoms scores and was significant *F*_2,87_ = 37.55 *p* < 0.0001 (Table 3; Figure 1).

**Table 3:** Mediation model of the mediating role of immature defenses in the relationship between guilt and psychological distress.

Variables		Overall mode fit			Significance of regression coefficient			
Outcome	Predictor	*R*	*R* ^2^	*F*	*B*	LLCI	ULCI	t
Immature defenses	Guilt	0.2463	0.0607	5.6845^*^	0.8262	0.1375	1.5148	2.3842^*^
Psychopathological symptoms	Guilt immature defenses	0.6806	0.4633	37.5483^***^	0.0185 0.0072	0.0105 0.0048	0.0265 0.0096	4.5811^***^ 6.0008^***^

*N = 90.*

* *p* < 0.05;

*** *p* < 0.001.

The overall model related to state shame accounted for 40.57% of the variance in psychopathological symptoms scores and was significant *F*_2,87_ = 29.69 *p* < 0.0001 (Table 4; Figure 1).

**Table 4:** Mediation model of the mediating role of immature defenses in the relationship between shame and psychological distress.

Variables		Overall mode fit			Significance of regression coefficient			
Outcome	Predictor	*R*	*R* ^2^	*F*	*B*	LLCI	ULCI	t
Immature defenses	Shame	0.2127	0.0453	4.1719^*^	0.7324	0.0198	1.4450	2.0425^*^
Psychopathological symptoms	Shame immature defenses	0.6369	0.4057	29.6951^***^	0.0140 0.0077	0.0054 0.0052	0.0226 0.0102	3.2440^**^ 6.1403^***^

*N = 90.*

* *p* < 0.05;

** *p* < 0.01;

*** *p* < 0.001.

Considering the results obtained by the analyses, all the following hypotheses have been accepted: (1) State guilt and shame scores related to personal stressful events are positively correlated with psychopathological symptoms; (2) State guilt and shame scores related to personal stressful events are positively correlated with immature defenses; (3) Immature defenses mediates the relationship between, respectively, state shame and state guilt with psychopathological symptoms.

## Discussion

In the present, the role that state shame and state guilt activations related to stressful experience has on psychopathological symptoms through defense mechanism was analyzed. As hypothesized, both state shame and state guilt were significantly correlated with psychopathological symptoms. This is consistent with a large body of research that highlighted the dysfunctional role of these two self-conscious emotions (Cavalera et al., 2017, 2018; Dorahy et al., 2017; Cândea and Szentagotai-Tătar, 2018) when they are related to unresolved experiences. A prolonged condition of maladaptive guilt related to previous transgressions or bad mistakes can imply disruptive consequences for mental health. Consistently with other data (Rugens and Terhune, 2013; Taihara and Malik, 2016), the present study evidenced that feelings of hyper-responsibility related to unresolved state guilty are related to psychopathological distress. These can be expressed through very different psychopathological symptoms that may be related to the externalization of blame, anger, hostility, or self-resentment usually related to maladaptive guilt (Taihara and Malik, 2016).

As expected, state shame showed the strongest correlations with GSI suggesting that the deep discomfort elicited by shame can have dramatic relations with psychological imbalances. As the earlier literature suggest (Tracy and Robins 2006; Dorahy et al., 2017; Cavalera et al., 2018) shame can be related to internalizing problems, interpersonal misunderstandings, depression, anxiety, and eating disorders. Therefore, as confirmed by the present data, unresolved shameful feelings related to stressful personal experiences can be related to poor psychological adjustment. It is possible that maladaptive role of state shame on mental wellbeing may be particularly disruptive when personal level of frustration discomfort is high (Tangney et al., 2014).

One other crucial finding is that the effect of both state shame and guilt activations on psychological distress is partially mediated by immature defenses. More in detail we proposed a model in which state shame and guilt activations elicited through an autobiographical emotional memory task influence psychological distress through the negative effect of primitive defenses. Our model explains that a substantial proportion of psychological distress is related to shame and guilt activations and evidenced that immature defenses partially mediated this association. A possible explanation of the present mediation model is that both shame and guilt convey crucial information for personal identities that may be hard to process.

When these experiences are perceived as unsustainable, they can be related to a less integrated system of attention, working memory inefficiencies, and heightened cognitive intrusions (Dalenberg et al., 2012; Dorahy et al., 2017; Cavalera et al., 2018). The use of immature defenses can be an involuntary way to manage the effects of such disruptive attentive consequences trying to express feelings of panic or urgency (f.e. through acting—out or projection) or trying to reduce the psychological impact of such maladaptive emotions (f.e. through dissociation or repression) by creating psychological distance. The present model expands the results of previous research (Rugens and Terhune, 2013; Dorahy et al., 2017) by highlighting the relationship between guilt and shame and not only dissociation but also with other immature defense mechanisms. Since mature defenses are an indication not only of the severity of psychopathology, but also a good predictor of therapeutic alliance and treatment response (Bond and Perry, 2004), an accurate evaluation of the individual defense profile could be useful for planning specific interventions during psychotherapy (e.g., interpretive vs. supportive interventions) in order to manage unresolved experiences of shame and guilt.

### Limitations

The current study is not without limitations. Firstly, participants might have imprecisely reported how they retrospectively felt in relationship with their personal experiences. In addition to that, data could have been influenced by individual skill in get involved into personal memory about the past. Although the sample was not collected in a clinical setting, specific information regarding the presence of emotional problems was not collected.

Further, the order in which different measures were proposed may have had an effect on the results obtained. More specifically, the emotional memory task may have influenced the compilation of the subsequent self-rating scale. As it was considered more complex, priority was given to the emotional memory task which was therefore proposed as the first measure to be assigned. To encourage greater freedom in selecting past memories, we did not ask for specific information with respect to the type of event chosen. Therefore, participants had the opportunity to choose experiences with different levels of magnitude (i.e., rape or failing an exam). Despite this aspect, the presence of the SSGS-8 allowed for the control of subjective emotional activation beyond the nature of the triggering event.

Another limiting aspect is that the present study focused specifically on state shame and guilt emotions and omitted to include additional emotional memory tasks triggering other negative emotional experiences. Future research may explore the role of state fear or rage on psychological imbalances.

## Conclusion

The current study extends earlier research on psychological imbalances by looking at the potential maladaptive role of shame and guilt for mental health. Our findings provide evidence for the role of shame and guilt on psychological symptoms considering these emotions from a situational perspective. Since most of the existing literature investigated psychological implications of personal proneness to shame and guilt, the present data offer key contributions related to shame and guilt in terms of state emotions.

An important aspect is that data showed a partial mediation of defense mechanisms suggesting that they accounts for some (but not for all) of the critical aspects related to the relationship between self-conscious emotions and psychological distress. It is possible that other variables mediate or moderate the relationship between these negative self-conscious emotions and psychological distress. The level of frustration or personal predisposition of internalizing/externalizing problems can be interesting factors that can be explored by future research (Taihara and Malik, 2016).

The present findings provide critical implications for psychotherapists and mental health workers. Managing unresolved experiences related to shame and guilt may become a key target in psychotherapy that could help patients break recursive thoughts and maladaptive defensive strategies (Houazene et al., 2021). Specific efforts can be addressed in understanding and accepting the reasons and the feelings underlying shameful and guilty experiences fostering a self-compassionate attitude (Goss and Allan, 2010). This may have important crucial consequences in the reduction of psychological symptoms (Kelly et al., 2017). A specific attention may be also directed to the use of primitive defense mechanisms. A deeper awareness of these involuntary strategies may help the patients to notice and prevent the recursive patterns highlighted by shame and guilt and deactivate their negative potential.

## Data Availability Statement

The raw data supporting the conclusions of this article will be made available by the authors, without undue reservation.

## Ethics Statement

The studies involving human participants were reviewed and approved by Commissione Etica Università Cattolica del Sacro Cuore di Milano. The patients/participants provided their written informed consent to participate in this study.

## Author Contributions

CC, PA, OB, and OO made substantial contributions to the conception or design of the work and to the analysis and interpretation of data for the work. CC and OO finally approved the version of the paper to be published. All authors contributed to the article and approved the submitted version.

## Funding

Università Cattolica del Sacro Cuore contributed to the funding of this research project and its publication.

## Conflict of Interest

The authors declare that the research was conducted in the absence of any commercial or financial relationships that could be construed as a potential conflict of interest.

## Publisher’s Note

All claims expressed in this article are solely those of the authors and do not necessarily represent those of their affiliated organizations, or those of the publisher, the editors and the reviewers. Any product that may be evaluated in this article, or claim that may be made by its manufacturer, is not guaranteed or endorsed by the publisher.
