# Supplementary material for: Do Immature Defense Mechanisms Mediate the Relationship Between Shame, Guilt, and Psychopathological Symptoms?
Source: Front Psychol. 2022 May 3;13:832237. doi: 10.3389/fpsyg.2022.832237 (PMC9113260; doi:10.3389/fpsyg.2022.832237)
Supplement: Supplementary file 1 [file Data_Sheet_1.PDF]

Supplementary Materials

Table S1. Male characteristics.

Variables	Mean/SD	Range	Kurtosis		Skewness	
			Statistical	Standard error	Statistical	Standard error
Age	24.28/1.62	20-28	0.986	0.688	0.279	0.350
State-Guilt	26.26/7.99	12-46	-0.329	0.688	0.006	0.350
State-Shame	30.39/7.93	13-48	-0.119	0.688	0.051	0.350
Psychopathological Distress	0.79/0.42	0.01-1.72	-0.471	0.688	0.582	0.350
Immature Defenses	12.21/2.09	8.64-17.36	-0.340	0.688	0.213	0.350

N=46

Table S2. Female characteristics.

Variables	Mean/SD	Range	Kurtosis		Skewness	
			Statistical	Standard error	Statistical	Standard error
Age	23/2.42	19-28	-0.465	0.702	-0.227	0.357
State-Guilt	26.8/10.44	12-46	-1.079	0.702	0.276	0.357
State-Shame	36.05/9.19	20-58	-0.461	0.702	0.445	0.357
Psychopathological Distress	1/0.48	0.14-2.27	0.193	0.702	0.664	0.357
Immature Defenses	12.21/2.09	8.64-17.36	-0.340	0.688	0.213	0.350

N=44
